# Burkitt lymphoma research in East Africa: highlights from the 9^th^ African organization for research and training in cancer conference held in Durban, South Africa in 2013

**DOI:** 10.1186/1750-9378-9-32

**Published:** 2014-09-11

**Authors:** Kenneth O Simbiri, Joshua Biddle, Tobias Kinyera, Pamela Akinyi Were, Constance Tenge, Esther Kawira, Nestory Masalu, Peter Odada Sumba, Janet Lawler-Heavner, Cristina D Stefan, Franco M Buonaguro, Detra Robinson, Robert Newton, Joe Harford, Kishor Bhatia, Sam M Mbulaiteye

**Affiliations:** 1 State University of New York (SUNY) Upstate Medical University, New York, NY, USA; 2 University of California at San Francisco, San Francisco, CA, USA; 3 EMBLEM Study, St. Mary's Hospital, Lacor, Uganda; 4 EMBLEM Study, Moi Teaching and Referral Hospital, Eldoret, Kenya; 5 EMBLEM Study, Moi University, Eldoret, Kenya; 6 EMBLEM Study, Shirati Health Education and Development (SHED) Foundation, Shirati, Tanzania; 7 Bugando Medical Center, Mwanza, Tanzania; 8 Kenya Medical Research Institute, Kisumu, Kenya; 9 Westat, Inc, Rockville, MD, USA; 10 University of Stellenbosch, Cape Town, South Africa; 11 Instituto Nazionale Tumori, Naples, Italy; 12 University of York, Heslington, York, United Kingdom/Medical Research Council/International Agency for Research on Cancer (IARC), Lyon, France; 13 National Institutes of Health/NCI/DCEG, 9609 Medical Center Dr, Rm. 6E118 MSC 9704, Bethesda, MD 20892-9704, USA

## Abstract

A one-day workshop on Burkitt lymphoma (BL) was held at the 9^th^ African Organization for Research and Training in Cancer (AORTIC) conference in 2013 in Durban, South Africa. The workshop featured 15 plenary talks by delegates representing 13 institutions that either fund or implement research on BL targeting AORTIC delegates primarily interested in pediatric oncology. The main outcomes of the meeting were improved sharing of knowledge and experience about ongoing epidemiologic BL research, BL treatment in different settings, the role of cancer registries in cancer research, and opportunities for African scientists to publish in scientific journals. The idea of forming a consortium of BL to improve coordination, information sharing, accelerate discovery, dissemination, and translation of knowledge and to build capacity, while reducing redundant efforts was discussed. Here, we summarize the presentations and discussions from the workshop.

## Introduction

On November 20, 2013, scientists from 13 institutions held a one-day pre-conference workshop during the 9^th^ African Organization for Research and Training in Cancer (AORTIC) conference in Durban, South Africa (Additional file 1: Table S1) [1]. The workshop was motivated by a positive response to a successful 3-hour satellite meeting organized by Epidemiology of Burkitt Lymphoma in East-African Children and Minors (EMBLEM) at the 8^th^ AORTIC conference in 2011 in Cairo, Egypt to highlight research sponsored by the Intramural Research Program of the National Cancer Institute (NCI) in East Africa and in Ghana. The workshop objectives were to highlight Burkitt Lymphoma (BL) research in East Africa, one of the regions with the highest recorded rates of BL, and highlight the seminal contribution of BL research to cancer research in general and in Africa in particular. The workshop aimed to increase awareness about ongoing funded BL research, including epidemiology, treatment, and immunology; and to highlight the importance of cancer registration, capacity-building, and scientific publication in BL research in East Africa. The workshop also provided a platform to discuss ways to improve collaboration. Here we summarize the presentations, discussions, and conclusions from the workshop.

### The EMBLEM study

**Figure 1:**
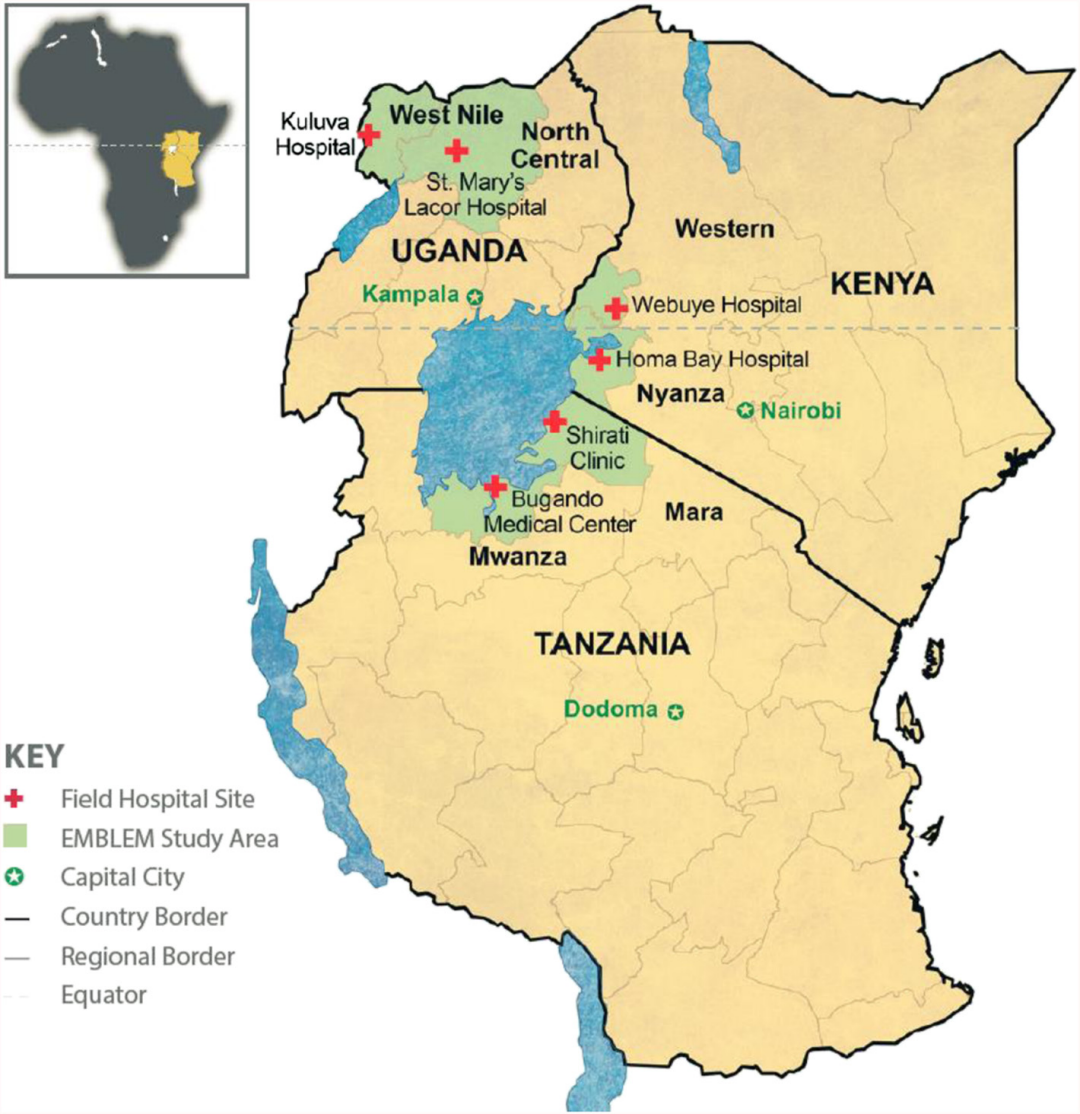
Map of East Africa showing six regions of the EMBLEM study area.

The workshop began with a presentation by Dr. Mbulaiteye about the objectives, design, and timeline of the EMBLEM Study (http://emblem.cancer.gov/). EMBLEM is a biomolecular-epidemiologic study funded by the Intramural Research Program of the National Cancer Institute (NCI) in collaboration with local investigators in Uganda, Tanzania, and Kenya (Figure 1). The objectives are to investigate the link between infections with *Plasmodium falciparum* (*Pf*) malaria, Epstein-Barr virus (EBV) and genetic risk factors with BL. Since BL is the most common childhood cancer, and *Pf* malaria, the putative cause, is the leading cause of morbidity and mortality in East Africa, findings from EMBLEM could influence the scope and direction of cancer research, care, prevention in East Africa. Historically, BL research in Africa has influenced oncology practice [2], including the first effective use of chemotherapy for BL, first demonstrated in the East Africa [3], and then used elsewhere and for other types of cancer [4]. Those early studies showed the value of combination therapy and CNS prophylaxis [5]. The uneven geographical patterns of BL, notably high incidence in low-lying humid areas in Africa suggested infectious etiology [6] and led to the discovery in 1964 of Epstein-Barr virus (EBV) [7] as the first human virus associated with a human cancer [8]. Today, EBV is designated as a Class 1 carcinogen for BL and is implicated in several other cancers [9]. EBV cooperates with co-factors, particularly *Pf* malaria [10], which was implicated based on correlational studies of the geographical distribution of *Pf* malaria with that of BL. Recent studies using anti-malaria antibody assays are providing strong evidence for a role of malaria in BL [11]. The results from these studies are difficult to interpret because antibody levels may change after disease onset (i.e., reverse causality bias). To overcome this limitation, some investigators have measured genetic polymorphisms in the hemoglobin gene that confer resistance to severe fatal forms of malaria among heterozygotes [12] to investigate the link between malaria and BL. Genetic polymorphisms are ideal because they are not subject to reverse causality bias and their distribution is governed by Mendelian randomization in the population [13]. Only three small studies, all conducted 40 years ago in Nigeria [14], Uganda [15] and in Ghana [16], have used this approach. They measured hemoglobin electrophoretic patterns for the sickle cell trait, which is associated with 70-90% protection from severe malaria [17], in cases and controls. In the study conducted in Nigeria, the frequency of the sickle cell trait in 95 BL cases was half that found in 320 age- and sex-matched hospital controls [14]. However, these results were not replicated in the other two studies conducted in Uganda (36 cases and controls) [15] or in Ghana (110 cases and 110 controls) [16] or in a recent study that was conducted in Kenya looking at 306 cases and 537 ethnically matched controls [18]. These inconsistent results do not invalidate the genetic approach because the sickle cell trait may account for only 2% of the malaria risk whereas genetic factors [19] account for 25% of malaria risk [20]. Thus, further studies are needed to evaluate effects of multiple genes simultaneously and also take into account epistatic (positive and negative) relationships between genes [19] to obtain valid results. One objective of EMBLEM is to enrol 1500 BL cases from six rural regions in East Africa where malaria transmission is holoendemic year round [21] and two healthy location-matched controls per BL case. The study will compare the cases and controls for 44 polymorphisms in 25 genes that are associated with *Pf* malaria resistance [21]. In secondary analyses, association of being a case with tag SNPs in the 25 genes will be evaluated to identify associations with non-candidate SNPs (SNPs with no association with malaria disease). Dr. Mbulaiteye noted replication of EMBLEM findings is needed and called for other studies to be conducted in similar settings, particularly in West Africa. He emphasized the importance of standardization of protocols through collaboration to accelerate the pace of discovery, replication, and translation, with the ultimate objective being to improve care and prevent BL.

Dr. Tobias Kinyera, EMBLEM Study Coordinator in Uganda, reviewed the recent epidemiology of BL in Uganda. He noted that the geographical patterns of incidence since 2000 mirror the historical patterns noted 40 years ago. The incidence was lowest (0.1 cases per 100,000 children) in Western Uganda and highest (2–15 cases per 100,000) in northern Uganda [22]. However, while BL in the 1960s was typically described as affecting jaws [23], the predominant presentation today was mostly abdominal with or without facial tumors [22]. Similar changes in anatomic site presentation have been reported in Ghana [24], but the reasons underlying these changes in predominate anatomic presentation are unknown. He speculated that greater availability of ultrasound, which provides more accurate information about anatomic site involvement in the abdomen than was possible using only clinical examination [25], might be responsible. Dr. Kinyera reported that the EMBLEM study is being implemented in northern Uganda and that the cases spotted in that region are referred to two local hospitals with the capacity to diagnose and treat BL. To increase case spotting in the region, EMBLEM has trained local health workers and community stakeholders to increase their awareness about BL and to encourage them to refer the cases to the study hospitals for biopsy, histological diagnosis and early treatment [26]. These procedures (spotting and referral) have been integrated within the local community health system (the main stakeholder) workflow. The procedures were leading to broad-based improvements in community engagement [21], diagnostic pathology [27], and consistent use of protocol-based treatment for BL [26]. In concluding remarks, Dr. Kinyera noted that basic laboratory upgrades implemented to facilitate research sample collection and long-term storage have enabled fieldwork to be conducted in the rural areas of northern Uganda. To date, 427 potential BL cases have been spotted and 220 of them have been enrolled into the study. During the same period, 1201 location-matched controls had been enrolled.

Dr. Esther Kawira, EMBLEM Study Co-PI in Tanzania, reported that infrastructure improvements similar to those described in Uganda had also been implemented to support EMBLEM scientific research in rural Tanzania. These include high-speed internet to support communication, data faxing and access to the EMBLEM share portal for secure transfer of sensitive research data, study protocol archives; basic research equipment such as ultra-low freezers, QBC hematology analyzers, and bench-top centrifuges. In addition, the staff was trained in good clinical and laboratory practice. Dr. Kawira noted that an analysis of historical BL data had revealed that BL incidence patterns (2000 to 2010) in northern Tanzania had not changed much from 40 years ago and the incidence varied from 2 cases per 100,000 in the Mwanza Region to 22 cases per 100,000 in Tarime in the Mara Region [28]. An unexpected finding was that the incidence of BL fell between 2005 to 2009, the reasons for which were not clear [28]. To date 298 potential BL cases have been spotted and 60 of these were eligible and have been enrolled. Control enrollment is scheduled to begin in 2014.

Dr. Constance Tenge, EMBLEM Co- PI in Kenya, reported on EMBLEM in Kenya. She noted that an environment of relative competition characterized the work in Kenya since there were several groups who had initiated or had plans to initiate BL studies in overlapping geographical areas but with little coordination across them. This has created unique challenges about how to present a simple and positive message about BL to the community. Consistent with the principle of community engagement [21,26], EMBLEM had established field sites at Homa Bay and Webuye District Hospitals, and established a contract with the Academic Model Providing Access to Healthcare (AMPATH) [29] at Moi Teaching and Referral Hospital (MTRH) to use their laboratories for long-term sample storage and another contract with MTRH for histological diagnosis of BL. To date, 258 potential BL cases have been spotted and 90 of these have been enrolled. Control enrollment is scheduled to begin in 2014.

Dr. Kishor Bhatia, EMBLEM Co-PI and adjunct Investigator in the Division of Cancer Epidemiology and Genetics at NCI, gave an overview titled “Molecular studies: What is possible within EMBLEM structure?” He noted that the design of EMBLEM to enroll a large number of cases from diverse geographical areas is an excellent platform for molecular studies to investigate host-pathogen interaction in BL carcinogenesis [30,31]. The use of biopsy confirmed cases and collection of residual tissue to verify the diagnosis of BL was a strength [27], which had already enabled EMBLEM to contribute to one study of functional genomics of BL [30]. Tissues from EMBLEM were used to confirm mutations affecting the transcription factor TCF3 (E2A) or its negative regulator ID3. These genes influence the signaling of the pro-survival phosphatidylinositol-3-OH kinase pathway that had been discovered through the study of BL tumor cell lines. These studies have indicated substantial differences in the percentage of mutated CCND3 by BL subtype (38% CCND3 in sporadic BL vs. 1.8% in endemic BL) [30]. These results pave the way to use high-resolution SNP arrays to study endemic BL as has been done in sporadic BL [30,32,33], and point to exciting possibilities for discovery of molecular abnormalities that initiate or drive malignant growth in African BL cases and the possible interaction with EBV [34]. Furthermore, high-resolution SNP array studies could shed light on the molecular heterogeneity of BL and suggest novel ways for molecularly targeted treatment of BL [2] or lead to discovery of non-human sequence data suggesting novel viral sequences or EBV variants that may be correlated with BL tumor development [2].

Dr. Peter Odada, Senior Researcher at KEMRI and EMBLEM Co-PI in Kenya, discussed the relationship between malaria and BL in Kenya [35]. Given the negative impact of malaria on health and nutrition, he speculated that micronutrient deficiency compounded by malaria infection might influence the risk of BL. In support of this idea, he shared the results from his recent study in Kenya showing that children living in high BL incidence areas were more likely to have lower Glutathione peroxidase (GPx) levels and higher EBV viral loads than children from low BL incidence areas [36].

Dr. Kenneth Simbiri, Instructor of Microbiology and Immunology at SUNY Upstate Medical University, presented a paper titled “Cancer in Africa in 21^st^ Century: Paradigm Shift”. He reviewed recent literature about associations between viruses and cancer and noted the unacceptably high burden of infection-associated cancers in Africa, including cervical, vulvar, conjunctival, and anal [8]. Given the overlap of high HIV rates in Africa with many virally associated cancers, he stressed the need for studies to be conducted about infectious agents causing cancer and utilizing that knowledge in cancer control efforts in Africa [37-39].

Dr. Juliana Otieno, Pediatrician and Chief Administrator of Jaramogi Oginga Odinga Teaching and Referral Hospital (JOOTRH) in Kisumu, Kenya presented a paper on Burkitt lymphoma incidence in Western Kenya: experience from JOOTRH. Dr. Ann Moormann, Associate Professor at the University of Massachusetts Medical School, Worcester, MA, USA and a Visiting Scientist at KEMRI, presented a paper on EBNA T cell responses in BL in Kenya. The details of these presentations are not included in this report as they contain unpublished data.

### Treatment of Burkitt lymphoma in South Africa

Dr. Cristina Stefan, Professor and Head of Pediatric Oncology, University of Stellenbosch presented her data on “BL in HIV positive children in South Africa during 1990 – 2008 from the South African pediatric cancer registry” [40,41]. Dr. Stefan reported that the survival rate for Hodgkin lymphoma was 45.8% in HIV positive children compared to a survival rate of 80% in HIV-negative children. Likewise, mortality in HIV positive children with BL was high due to toxicity of treatment, and up to 10% could not be treated because of unstable clinical conditions. Dr. Stefan noted that although all cases of BL in South Africa were histologically, cytogenetically, or immunohistochemically tested, there was still a need for consistency across diagnostic types since there is no “gold standard” for BL diagnosis [27]. She expressed willingness to share tissues from her cases for genetic studies of BL.

### Cancer registration as a catalyst for research in Burkitt lymphoma

Dr. Robert Newton, who has longstanding research experience conducting cancer research in sub-Saharan Africa and is the representative of the International Agency for Research on Cancer (IARC) Global Initiative for Cancer Registration in Africa, presented an overview about the role of cancer registries in cancer research. Focusing on BL as a model disease, he illustrated the challenges of cancer registration. He noted that the first challenge for cancer research and registration is defining the cancer of interest using reproducible criteria. Histology is the typically accepted standard for diagnosis of cancer, including of BL, but the lack of high-quality histology in Africa undermines the confidence in African cancer data [27,42]. Improvements in the quality of formalin-fixed and formalin-embedded tissues would enable the use of immunohistochemistry and cytogenetics for diagnosis, particularly of BL, and improve confidence in the data. The second challenge is identifying all the cases of cancer that occur in a well-defined population. To generate accurate cancer statistics (incidence and mortality), the population at risk that is covered by a particular registry needs to be defined, and all the cases occurring in the population need to be identified. Both definition of population at risk and obtaining complete cancer data are difficult to achieve in Africa given the lack of accurate census data for most countries [43,44], and the low level of service provision or access. Together, these difficulties introduce uncertainty in cancer statistics from Africa. Dr. Newton noted that registration of childhood cancers, which are rare and often present as look-alikes to many common childhood conditions, such as jaw or abdominal swellings and anemia in the case of leukemia, could be even more challenging. Over or under-diagnosis could have a large impact on estimation of the incidence of rare cancers. On the question of whether to establish a BL registry, Dr. Newton recommended establishing or strengthening general cancer registries to register all cancers, including those in children. A general cancer registry would facilitate the conduct of comparative studies of different cancers and studies of cancer-specific trends. Since BL in Africa is strongly associated with *Pf* malaria, cancer registry studies could be used to monitor BL trends in relation to interventions to prevent *Pf* malaria in holoendemic areas and thereby provide indirect evidence for the association between *Pf* malaria and BL.

### Opportunities for African scientists to publish

On behalf of Dr. Franco Buonaguro, Editor-in-Chief of *Infectious Agents and Cancer* journal (http://www.infectagentscancer.com/), Dr. Mbulaiteye described the importance of scientific publication and encouraged African scientists to take advantage of open-access journals. He explained that *Infectious Agents and Cancer* journal has prioritized papers reporting data on infectious agents that cause or are linked to cancer. This focus is important for Africa where up to one-third of cancers are caused by infections [45]. Moreover, the open access policy guarantees that published research can be accessed immediately via any internet connection. To increase access to authors from low and middle income countries (LMICs) and sharpen focus, the journal has added sections on Clinical Oncology [46] and Cancer Centers in Resource Limited Settings [47]. In addition, the journal routinely waives Article Publishing Charges for up to six articles submitted by scientists from LMICs who lack grant support for their publication. Finally, he reported that Thomson Reuters are tracking the journal citations for “Impact Factor” assessment with expected publication of results in June 2014. This should further strengthen the mission of *Infectious Agents and Cancer*.

### Capacity building for cancer research in Africa

**Figure 2:**
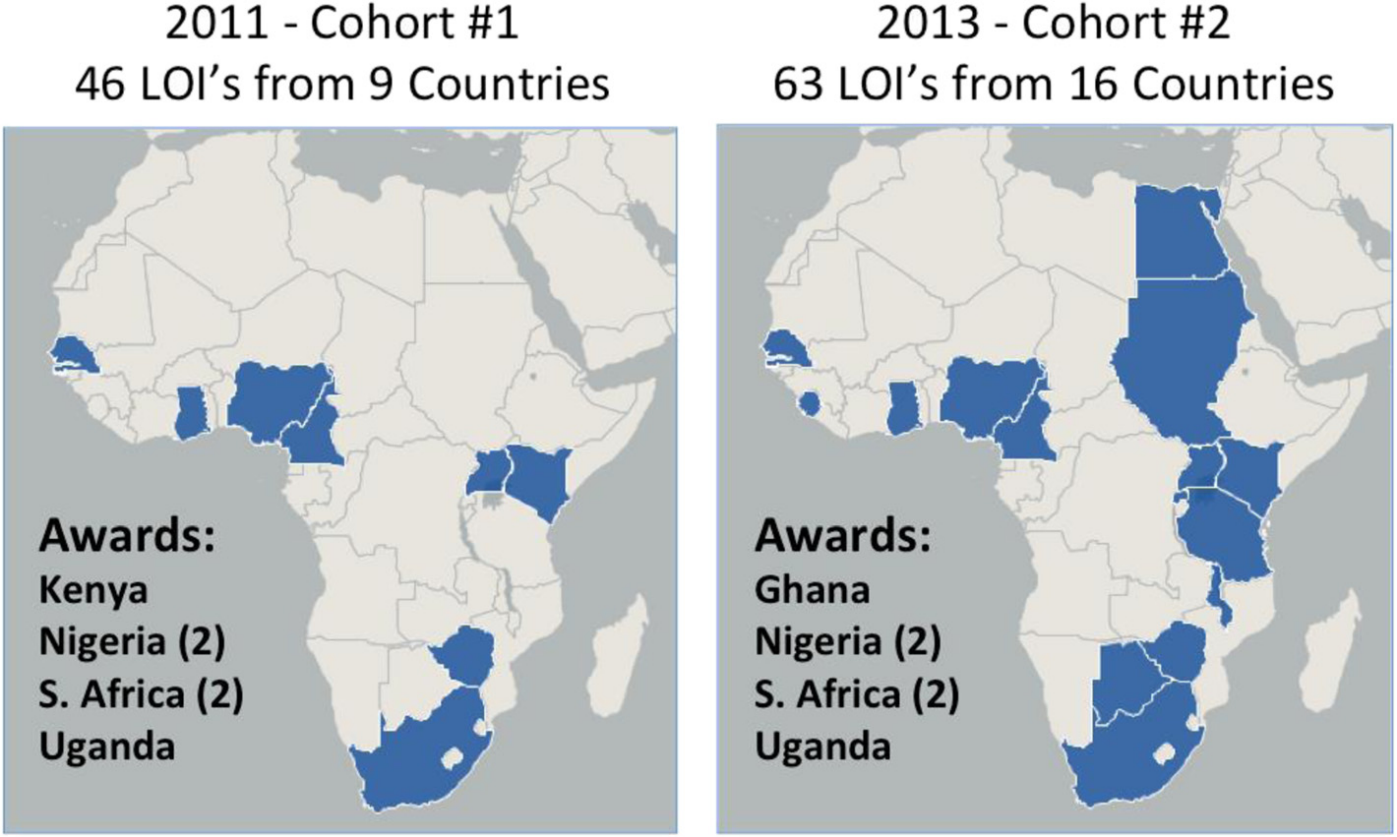
**Maps showing countries from which Letters of Intent (LOI’s) were received for Beginning Investigator Grants for Catalytic research (BIG Cat).** The BIG Cat initiative is overseen by the African Organization for Research and Training in Cancer (AORTIC) with funding to date having been provided by the U.S. National Cancer Institute. The application process involves submission of a LOI followed by a request for and submission of a full project proposal. One review criteria for applications is relevance to the cancer burden of Africa. Between Cohort #1 (2011) and Cohort #2 (2013), awareness of this funding opportunity increased as reflected in the number of LOI’s received as well as countries represented by the applicants.

Dr. Joe Harford of the NCI presented a talk entitled “Capacity Building in Africa” that outlined concrete ways the NCI is supporting cancer research and prevention in Africa [48]. To overcome the hurdle of preliminary data from a grant provider’s point of view, which is critical for funded grants, the NCI has funded through AORTIC “Beginning Investigator Grants for Catalytic (BIG CAT)” Research in 2010 to support young researchers in Africa to conduct studies that could lead to grant applications by African scientists [26]. Dr. Harford noted that BIG CAT grants have no categorical exclusions on the type of cancer research performed (bench, clinical, or epidemiological), but the projects should have a short lifespan (two years) and should be preliminary in nature. To date, 12 two-year BIG CAT grants have been awarded to African scientists (Figure 2). The NCI also supports a four-week Summer Cancer Prevention Fellowship each year in Bethesda, Maryland, where African scientists (alongside scientists from all over the world) can learn about cancer research methods. To date, 118 researchers from Africa have attended this course, including five who are working with EMBLEM or affiliated institutions. Finally, the NCI is funding pilot collaborations between US based cancer centers and their collaborators in LMICs via supplement to the P30 core grant of the US based institution.

### Future directions: a consortium of Burkitt Lymphoma studies in Africa

Ms. Detra Robinson from Westat, in Rockville, MD, discussed the challenges of studying BL, which is a rare condition. For example, despite the substantial amount of data reported at the meeting, one of the striking limitations highlighted was small sample size to obtain robust results. Other limitations were use of non-uniform methods of case spotting sample collection, diagnosis and treatment. All these limitations complicate meaningful comparisons within and across studies. She noted that the model adopted by EMBLEM to link multiple sites within and across several countries is worth considering and, if possible, expanding. Citing experiences from developed countries, where large-scale international collaborations have been established to study rare diseases, Ms. Robinson advanced the idea that the limitations noted above could be minimized by forming a consortium of BL research in Africa. Consortia provide a platform for long-term, large, well-powered studies. The successful establishment of EMBLEM in three East African countries increases our confidence that this type of consortium is possible in Africa. The consortium would reduce costs of research by reducing duplication, redundancy, and it would support linking clinical and basic immunology or molecular studies to the ongoing epidemiological work. Notwithstanding the challenges of forming a consortium, including obtaining funding, negotiating intellectual property and publication rights, and country specific-regulations, the benefits could be enormous.

### Panel discussion

The session ended with a lively panel discussion that included, among others, Drs. Harford, Newton, Bhatia and Mbulaiteye, and was moderated by Dr. Simbiri. Discussion included governance of a BL consortium, the research questions the consortium would address, and advantages of a consortium versus single-center studies. The multifactorial etiology of BL and its rare occurrence ensures that no single study will have sufficient expertise or power to address all the important questions. A consortium approach would enable faster integration of findings by providing efficient and cost-effective sharing mechanisms of research data and samples to a group of otherwise independent scientists from varying disciplines. Panel members sought to distinguish between the capability of collecting data and the necessity for data uniformity. The issue of coordinating multi-country ethical review board approvals and risks of delays in implementation and amendments of protocols was raised. Dr. Harford pointed out that EMBLEM is a mini-consortium and its experience of obtaining multi-country ethical review board approvals suggests that ethical approval and protocol coordination at a consortium level is possible. He concurred that governing procedures need to be established early in to manage expectations and minimize conflict. Dr. Mbulaiteye explained how EMBLEM has attempted to standardize research methods across the collaborating sites and he agreed that obtaining ethical approvals and coordinating protocol implementation across multiple sites is a challenge that must (and could) be overcome. Consortii provide a cost-effective platform for discovery, including in sub-group analyses. With few opportunities to replicate findings within a single study, a consortium would provide a structure for combining many small studies with limited available data and biospecimens. The consortium could borrow established procedures for sharing data and samples and the governance from successful consortia

Dr. Bhatia and Dr. Newton encouraged the BL investigators to consider extending the consortium to groups outside Africa, such as Brazil, India, or Papua New Guinea, who have similar or related research interests. Dr. Newton noted that the key to collaboration is a willingness to engage and that was necessary to address the difficulties of establishing a consortium. If there is an initial willingness to work together, there can be a consortium. The sentiment was that there will be challenges at the beginning, but the objective of forming a consortium is attainable. The critical issues to be resolved were governance structure, funding, and harmonization of objectives. On the question of a BL specific consortium versus an all cancer consortium, Dr. Harford suggested staying focused and limiting the consortium to BL. Furthermore, he encouraged the researchers to define metrics for assessing success, e.g., establishing a bioinformatics infrastructure or a tissue repository, such as is being done for the Human Heredity and Health (H3) African project and nurturing and supporting investigators to obtain independent funding, such as from the BIG CAT grants.

## Conclusion

The main outcomes of the meeting were improved sharing of knowledge and experience about ongoing epidemiologic BL research, BL treatment in different settings, the role of cancer registries in cancer research, and opportunities for African scientists to publish in scientific journals. The idea of forming a consortium of BL to improve coordination, information sharing, accelerate discovery, dissemination, and translation of knowledge and to build capacity, while reducing redundant efforts, was discussed.

## Competing interests

The authors of this paper declare that they have no financial commitment to any organization. The authors do not hold any stocks or shares in an organization that may in any way gain or lose financially from the publication of this manuscript, either now or in the future. The authors do not hold or are currently applying for any patents relating to the content of the manuscript nor have received reimbursements, fees, funding, or salary from an organization that holds or has applied for patents relating to the content of the manuscript. The authors have no other financial competing interests or non-financial competing interests (political, personal, religious, ideological, academic, intellectual, commercial or any other) to declare in relation to this manuscript. The opinions in the manuscript do not necessarily reflect the views of the National Cancer Institute, or the National Institutes of Health, Department of Health and Human Services or the United States Government.

## Authors’ contributions

SMM conceived the idea, KOS and SMM facilitated the meeting, and JB took notes during the meeting and drafted the manuscript with KOS. TK, EK, PAW, NM, POS, CT, JLH, CS, FB, DR, RN, JH, KB made oral presentations, discussed and interpreted data. All of the authors read, edited, and approved the final draft of the manuscript.

## Supplementary Material

Additional file 1 **9th International conference of aortic emblem.** Selected NCI funded reasearch on Burkitt lymphoma. **Table 1:** Agenda for the EMBLEM pre-conference workshop held at the 9^th^ African Organization for Research and Training in Cancer in Durban South Africa. Click here for file
