# Supplementary material for: Burkitt lymphoma research in East Africa: highlights from the 9th African organization for research and training in cancer conference held in Durban, South Africa in 2013
Source: Infect Agent Cancer. 2014 Sep 11;9:32. doi: 10.1186/1750-9378-9-32 (PMC4163050; doi:10.1186/1750-9378-9-32)
Supplement: Additional file 1 — 9th International conference of aortic emblem. Selected NCI funded reasearch on Burkitt lymphoma. Table 1: Agenda for the EMBLEM pre-conference workshop held at the 9th African Organization for Research and Training in Cancer in Durban South Africa. [file 1750-9378-9-32-S1.docx]

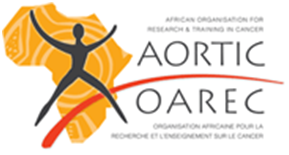

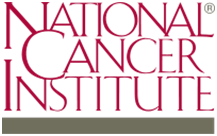


**9th International Conference of AORTIC EMBLEM**

**SELECTED NCI FUNDED REASEARCH ON BURKITT LYMPHOMA:**

**November 20, 2013 9:00am—5:30pm**

**Convener:** Dr. Sam M. Mbulaiteye, M.D. National Cancer Institute Bethesda, Maryland, USA *mbulaits@mail.nih.gov*

**Session co-chair:** Dr. Kenneth Simbiri, Ph.D. SUNY Upstate Medical Syracuse, New York, USA *simbirioa@gmail.com*

**Session co-chair:** Kishor Bhatia, PhD. National Cancer Institute Bethesda, Maryland, USA *bhatiak@mail.nih.gov*

**EMBLEM (Chair: Kishor Bhatia, NCI)**

**0900-0915: Sam Mbulaiteye (NCI, USA)**

*Collaboration: Objectives, design, and timelines*

**0920-0935: Martin Ogwang (St. Mary’s Hospital, Lacor)**

*Status update in Uganda*

**0940-0945: Esther Kawira (SHED Foundation)**

*Status update in Tanzania*

**0950-1005: Constance Tenge (Moi University)**

*Status update in Kenya*

**1010-1025: Kishor Bhatia (NCI, USA)**

*Molecular Studies*

**Burkitt Lymphoma Treatment & Consortia Development**

**1530-1545: Fred Okuku (Uganda Cancer Institute)**

*Overview of current status of Burkitt lymphoma treatment in Africa*

**1550-1605: Ann Moormann (University of Massachusetts)**

*Burkitt lymphoma in Western Kenya: A prospective survival study from 2003-2012*

**1610-1625: Cristina Stefan (University of Stellenbosch)**

*Burkitt lymphoma treatment in South Africa*

**1630-1700: Chris Williams (University of Washington Seattle)**

*Capacity Building in Hematology/Oncology in African Countries*

**1700-1720: Detra Robinson (Westat)**

*Opportunity for forming a Consortium on Burkitt lymphoma in Africa*

**National Cancer Institute Funded**

**1100-1115: Juliana Otieno (Teaching and Referral Hospital, Kisumu) *junyarchiga@gmail.com***

*Burkitt lymphoma incidence in Western Kenya: experience from JOOTRH*

**1120-1135Ann Moormann (University of Massachusetts) *ann.moormann@umassmed.edu***

*EBNA T cell responses in Burkitt lymphoma in Kenya***1140-1255: Odada Sumba (KEMRI)**

*Burkitt lymphoma incidence in Nyanza Province—role of malaria*

**1200-1215: Kenneth Simbiri (SUNY)**

*Dealing with Cancer in Africa in the 21st Century: A paradigm shift*

**1220-1335: Franco Buonaguro (Infectious Agents and Cancer)**

*Opportunities for African Scientists to publish on the link between cancer and infections*

**1240-1245: Joe Harford (NCI, Bethesda, Maryland)**

*Capacity Building: Catalytic Initiatives by NCI*

**Burkitt Lymphoma Registry and Pathology**

**1430-1455: Robert Newton (Research Council/IARC)**

*Overview of comprehensive population-based BL registration in the Africa Cancer Registry Network*

**1500-1525: Leona Ayers (Ohio State University)**

*Accuracy of histopathology for epidemiology and clinical studies*
